# Association of dementia comorbidities with caregivers’ physical, psychological, social, and financial burden

**DOI:** 10.1186/s12877-023-03774-9

**Published:** 2023-01-31

**Authors:** Jingyi Zhang, Jing Wang, Hua Liu, Chenkai Wu

**Affiliations:** 1 Dartmouth College, Hanover, NH USA; 2 School of Nursing, Fudan University, Shanghai, China; 3 Department of Neurosurgery, The Affiliated Kunshan Hospital of Jiangsu University, Suzhou, China; 4 Global Health Research Center, Duke Kunshan University, Kunshan, China

**Keywords:** Dementia caregiving, Chronic conditions, Comorbidity, Caregiving burden, Caregiving gain

## Abstract

**Background:**

Informal caregivers of older adults with dementia may experience substantial burdens during their caregiving process, especially when caring for older adults with other comorbid conditions. This study evaluated whether and how comorbidity burden for persons with dementia (PWD) was associated with caregivers’ physical, psychological, social, and financial burden as well as caregiving gain.

**Methods:**

Data were from 1,065 community-dwelling older adults living with dementia and their primary caregivers in the National Health and Aging Trends Study and the National Study of Caregiving. PWD’s comorbidity burden was measured by the count of chronic conditions and the pattern of comorbidity identified by the latent class analysis (LCA). We considered four domains of caregiving burden—physical, psychological, social, and financial burden. We used linear regressions to identify the unadjusted and adjusted associations between PWD’s comorbidity burden and caregiving burden and gain.

**Results:**

Of 1,065 PWD, 13.5% had 0–1 and 24.9% had 5 or more number of comorbid chronic conditions, respectively. After multivariable adjustment, an additional chronic condition is associated with an 0.11- and 0.36-point increase in caregivers’ physical and psychological burden, respectively. Caregivers of PWD with 5 or more chronic conditions had a 0.64- and 2.22-point higher score of physical and psychological burden, respectively, than those caring for PWD with 0 or 1 comorbid condition. LCA divided PWD into two classes, a high comorbidity class (69.0%) and a low comorbidity class (31.0%). Caregivers of PWD in the high comorbidity burden class had a 0.46-point higher score of physical caregiving burden than those in the low comorbidity burden class. No significant association was found between care recipients’ comorbidity burden and their caregivers’ social and financial burden or caregiving gain.

**Conclusions:**

The comorbidity burden of PWD was associated with their caregivers’ physical and psychological caregiving burden. Relevant interventions to manage the comorbid conditions of people living with dementia and support their caregivers are crucial to improving their physical health and psychological wellbeing.

**Supplementary Information:**

The online version contains supplementary material available at 10.1186/s12877-023-03774-9.

## Background

Persons with dementia (PWD) bear severe deteriorations in their memory, learning, or other cognitive functions that substantially interfere with their everyday living, level of independence, and communication capabilities [1]. Current treatments for dementia only show temporary and limited effects on managing the PWD’s symptoms and behavioral problems [2, 3]. Consequently, dementia demands a considerable amount of care from the PWD’s caregivers, and caring for PWD can be substantially stressful [4, 5]. As PWD greatly depend on others in their daily activities and face unique behavioral and psychological challenges, additional burdens are added to their informal caregivers in a number of ways, including physical strain, emotional stress, social isolation, and financial hardship [6]. In addition to caregiving burden, studies have also shown that dementia caregivers simultaneously experience significant gain through personal and spiritual growth, increase in their sense of mastery [7], and a greater and more positive appreciation of life [8]. Dementia caregiving is thus a multidimensional experience that involves both substantial burden and growth.

The health and caregiving of PWD become even more complicated when PWD have the simultaneous presence of other chronic conditions. For PWD, comorbidities are associated with many adverse consequences, such as increased healthcare utilization and cost [9], increased emergency department visits [10], and poor coordination between healthcare providers in different specialties that comes from a lack of medical training in caring for comorbid PWD among specialized healthcare providers [11]. The poor quality of healthcare that PWD with comorbid conditions receive can further increase the load of care that their informal caregivers have [12]. Due to PWD’s impaired cognitive functions as well as healthcare providers’ and caregivers’ lack of person-centered, effective communication with PWD, individuals with dementia tend to have poor understanding of their own conditions; therefore, it is especially difficult for caregivers to manage with and care for PWD with other simultaneous chronic conditions, such as diabetes, cancer, arthritis, or other conditions that also require a significant amount of care [13]. Caregiving for PWD with comorbid conditions can therefore become a significantly burdensome and overwhelming experience with lowering gain [12, 14].

Previous studies have shown that more comorbid conditions were associated with a higher overall burden for caregivers of PWD [15, 16]. Certain comorbidities can also lead to a decrease in caregiving gain [12]. In those studies, however, caregiving burden is often times measured by a single score that fails to reflect the different domains of caregiving burden that a caregiver might experience when caring for PWD. In fact, the burden from caregiving can come from different sources and have different manifestations. For example, physical burden may come from exhaustion and disruption of sleep. Emotional burden may come from the uncertainty of disease progression or the anxiety from PWD’s behavioral and psychological symptoms. Social burden may come from sacrificed time spent with families, friends, or other relationships. Financial burden may come from a reduction in caregiver’s work hours or a loss of their sources of income [5, 17]. Therefore, it is important to capture the complexity of caregiving burden and gain when studying how comorbidities impact a caregiver’s experience in each of these different domains.

The aim of this study is thus to evaluate whether and how comorbidity burden for older adults with dementia is associated with their caregiver’s physical, psychological, social, and financial burden as well as caregiving gain.

## Methods

### Data and participants

The National Health and Aging Trends Study (NHATS) is an ongoing, longitudinal study that surveys a nationally representative sample of Medicare beneficiaries ages 65 years and older in the United States [18]. All participants gave informed consent and ethical approval was obtained. The National Study of Caregiving (NSOC) studies family and other unpaid caregivers to older persons living with limitations in daily activities, which had been conducted three times in conjunction with the NHATS [19].

We used data from the 2011, 2015, and 2017 waves of the NHATS and NSOC. We did not include data from other waves because the NSOC surveys were only distributed to caregivers in 2011, 2015, and 2017. A total of 12,427 care recipients participated in the 2011, 2015, 2017 waves of the NHATS and 3,778 caregivers were surveyed in NSOC. Our analytic sample comprised of 1,065 community-dwelling older adults with probable dementia and their caregivers. A care recipient is counted as probable dementia if they 1) had self- or proxy-reported diagnosis; 2) met the AD8 diagnosis criteria for dementia based on their orientation, executive function, and memory assessments [20]; or 3) scored more than 1.5 standard deviations below the mean in two or more cognitive testing domains. For care recipients with multiple caregivers participating in the NSOC, we selected their primary caregiver who had the most caregiving hours in the last month prior to their interview [21]. For caregivers who were surveyed in multiple waves of NSOC, we used data from the first wave.

To account for oversampling and non-response bias in the complex survey design of NHATS and NSOC, we used the NSOC cross-sectional weight in all models to make the caregiver sample nationally representative.

### Comorbid chronic disease burden

Nine comorbid chronic conditions were measured by NHATS and considered in this study: heart disease (heart attacks, myocardial infarction, angina or congestive heart failure, and other heart diseases), hypertension, arthritis, osteoporosis, diabetes, lung disease, stroke, cancer, and depressive symptoms. Having depressive symptoms was determined based on the Patient Health Questionnaire-2 (PHQ-2) screening test, which inquires about the frequency of depressed mood over the past two weeks [22]. Each participant was asked, “Over the last month, how often have you a) had little interest or pleasure in doing things; b) felt down, depressed, or hopeless?” A response of “Not at all”, “Several days”, “More than half the days”, and “Nearly every day” was coded 0, 1, 2, and 3, respectively. The PHQ-2 score ranges from 0–6. A person with a score of 3 or greater was considered having major depressive disorder. All other chronic conditions were self- or proxy-reported.

### Caregiving burden and gain

We considered four aspects of caregiving burden: physical, psychological, social, and financial burden. Each aspect was assessed by a composite score calculated based on multiple self-reported items capturing caregiving experience. Physical burden includes five items: whether the caregiver is exhausted when go to bed at night and whether their activities are limited by pain, breathing problems, low strength, or low energy (1 = very much, somewhat). Psychological burden includes eighteen items: whether the caregiver has anxiety or depression, which are determined based on Generalized Anxiety Disorder 2-item (GAD-2) and PHQ-2 criteria (1 = Yes) [23, 22]; has more things to do than they can handle, doesn’t have time for themselves, or needs change as soon as they get a routine going (1 = very much, somewhat); has a life with meaning and purpose, feels confident and good about themselves, likes their living situation very much, has an easy time adjusting to changes, or gets over illness and hardship quickly (1 = disagree somewhat, disagree strongly); gives up trying to improve their life a long time ago, or feels lonely because they have few close friends (1 = agree somewhat, agree strongly); feels bored, lonely, upset (1 = some days, most days, everyday); or feels cheerful, calm and peaceful, or full of life (1 = rarely, never). Social burden includes five items: whether caregiving keeps them from visiting families or friends, attending religious services, going out for enjoyment, volunteering, or caring for someone else (1 = Yes). Financial burden includes two items: whether caregiving keeps them from working for pay or made it harder for them to get work done in the past month (1 = Yes). Composite scores were calculated for each of the four domains, resulting in a physical burden composite score ranges from 0 to 5, psychological burden composite score ranges from 0 to 18, social burden composite score ranges from 0 to 5, and financial burden composite score ranges from 0 to 2.

We also considered caregiving gain, which includes four items: whether caregiving makes them more confident in abilities, teaches them to deal with difficulties, brings them closer to the care recipient, or gives them the satisfaction that the care recipient is well cared for (1 = very much, somewhat). A composite score for caregiving gain was calculated and ranges from 0 to 4.

### Covariates

Care recipients’ background characteristics include their gender (1 = female), age (65–74 years old, 75–84 years old, 85 + years old), and race (white non-Hispanic, Black non-Hispanic, Other non-Hispanic, and Hispanic). Caregivers’ background characteristics include gender (1 = female), age (1 = over 65 years), race (white non-Hispanic, Black non-Hispanic, Other non-Hispanic, and Hispanic), relationship to care recipient (1 = spouse, 0 = non-spouse), education level (1 = college or above), total caregiving hours in the past month, and whether the caregiver is involved in long-term caregiving for over five years (1 = Yes). We also adjusted for the type of caregiving assistance that the caregiver provides for their care recipients, including assistance with activities of daily living and instrumental activities of daily living (ADL-IADL), health managements, and medical tasks. The formal and informal caregiving support that caregiver received are also included as covariates.

### Statistical analysis

We described the sociodemographic characteristics of care recipients, caregivers, and caregiving burden by the year the care recipient was surveyed (2011, 2015, 2017) and the number of comorbid chronic conditions the care recipients have (0–1, 2, 3, 4, 5 or more). We used means and standard deviations for continuous variables and counts and percentages for categorical variables.

Care recipients’ comorbidity burden was measured in two ways: (1) count of chronic conditions and (2) pattern of comorbidity identified by the latent class analysis (LCA). For count of chronic conditions, we calculated the total number of comorbid chronic conditions the care recipient has in addition to dementia out of the nine conditions we considered. We modeled the count of comorbidities both continuously and in categories (0–1, 2, 3, 4, and 5 or more). For the LCA analysis, we identified patterns from the nine comorbid chronic conditions by examining two to five comorbidity burden classes and selected the optimal number of latent classes based on statistical criteria and interpretability. After we determined the final LCA model, we assigned each participant to a comorbidity burden class based on the highest estimated probability of class membership.

We used a series of linear regressions to identify the unadjusted and adjusted association between the count of comorbidities among PWD and their caregivers’ physical, psychological, social, and financial burden separately, as well as caregiving gain. After checking the residual plot, we decided to use the Poisson regression model for the physical burden outcome. We used the three-step approach to determine the association between LCA class membership with each distal outcome (e.g., physical burden). Care recipient’s gender, age, and race, as well as caregiver’s gender, age, race, relationship to care recipient, education level, caregiving hours, long-term caregiving status, caregiving activities (helping care recipient with ADL-IADL, health management, medical tasks), and formal and informal caregiving support were included as covariates in the multivariable adjusted models.

Descriptive analysis and linear regression models were carried out using STATA/SE, version 16.1 [24], and LCA was carried out using Mplus, version 8.7 [25].

## Results

### Sample description

Of the 1,065 care recipients in the weighted sample, 63.2% were women; 14.3%, 40.9%, and 44.8% were 65–74, 75–84, and 85 + years old respectively (Table 1). On average, care recipients had 3.33 (SD = 1.65) chronic conditions; 13.5%, 17.8%, 22.5%, 21.3%, and 24.9% had 0–1, 2, 3, 4, and 5 or more comorbidities, respectively. Of the 1,065 corresponding caregivers, 68.7% were women and 35.7% were over 65 years old; 19.1% were spouse of the care recipient, and 24.9% has a college or higher education degree (Table 1). 41.8% care recipients and their caregivers were interviewed in 2011, 30.6% in 2015, and 27.6% in 2017 (Supplementary Table S1). Chi-square test showed no significant differences in the care recipients or caregivers’ characteristics across these three years (Supplementary Table S1).

**Table 1:** Weighted sample characteristics of care recipients and caregivers by PWD’s number of comorbidities (weighted *N* = 1,065)

Characteristics	Count (%)
*Care recipients*	
Number of comorbidities	
0–1	148 (13.5)
2	179 (17.8)
3	254 (22.5)
4	227 (21.3)
5 or more	259 (24.9)
Sex, female	688 (63.2)
Race	
White, non-Hispanic	609 (68.0)
Black, non-Hispanic	334 (12.9)
Other, non-Hispanic	26 (5.47)
Hispanic	84 (13.6)
Age	
65–74 years old	97 (14.3)
75–84 years old	379 (40.9)
85 + years old	591 (44.8)
*Caregivers*	
Sex, female	797 (68.7)
Age, over 65 years old	399 (35.7)
Race	
White, non-Hispanic	328 (68.4)
Black, non-Hispanic	176 (13.7)
Other, non-Hispanic	15 (4.10)
Hispanic	49 (13.8)
Relationship to PLWD, spouse	205 (19.1)
Highest education, college or above	274 (24.9)
Self-rated health, poor or fair	235 (20.5)

*Note.* Count refers to the observed, unweighted counts. The total count in the unweighted sample is 1,067. % refers to percentages in the weighted sample

### Association between count of comorbid conditions and caregiving burden and gain

**Fig. 1:**
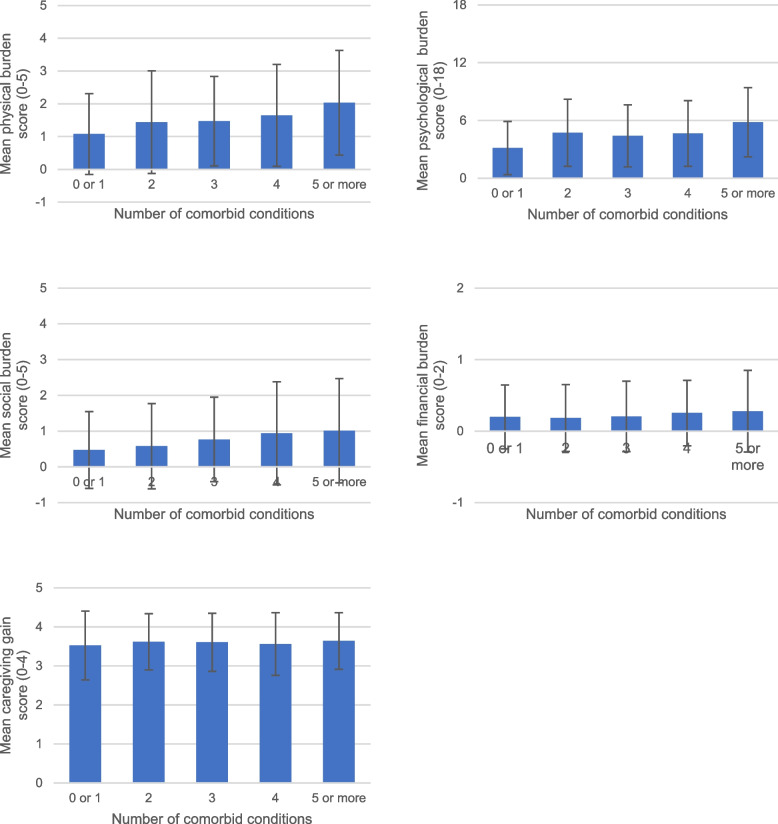
Mean caregiving burden and gain scores across disease burden groups. Note. In each histogram, a mean composite score of caregivers’ physical, psychological, social, and financial burden or their caregiving gain was plotted against the number of comorbid chronic conditions that care recipients have. Error bars show the mean ± 1 standard deviation

There were significant differences in the caregiving burden of PWD with different number of comorbidities (Fig. 1, Table 2). After multivariable adjustment, an additional chronic condition was associated with an 0.11-point increase in caregivers’ physical burden and an 0.36-point increase in caregivers’ psychological burden (Table 3). Caregivers of PWD with the highest comorbidity burden (5 or more chronic conditions) had a 0.64-point higher score of physical burden than those caring for PWD with 0 or 1 comorbid condition, as well as a 2.22-point higher score of psychological burden than those caring for PWD with 0 or 1 comorbid condition (Table 3). Poisson regression showed that after multivariable adjustment, for a one-point increase in the number of comorbidities, the difference in the logs of expected physical burden score is expected to increase by 0.06 (Table 4). However, the number of chronic conditions that the care recipient had was neither significantly associated with the social or financial burden that their caregivers bear, nor their caregiving gain after multivariable adjustment (Table 3).

**Table 2:** One-way ANOVA comparison of mean outcome scores across comorbid disease burden groups

Outcomes	*P*-value
Physical Burden	<0.0001 ^*^
Psychological Burden	<0.0001 ^*^
Social Burden	0.0001 ^*^
Financial Burden	0.2603
Caregiving Gain	0.8664

*Note*. One-way ANOVA comparison of mean was run for each of the five outcome scores across disease burden groups in the weighted sample. Results showed that there were significant differences between physical burden, psychological burden, and social burden scores across PWD groups with different number of comorbidities. There were no significant differences between financial burden or caregiving gain across comorbid disease burden groups

^*^ *p* < 0.05

**Table 3:** Weighted association between care recipient’s chronic disease burden and caregiver’s burden

	Beta coefficients (95% CI)	
	Unadjusted model	Adjusted model ^a^
***Outcome 1: Physical burden***		
Count of chronic diseases (0–9)	0.18 (0.12, 0.23) ^*^	0.11 (0.03, 0.20) ^*^
Disease burden		
0–1	Ref	Ref
2	0.36 (0.04, 0.69) ^*^	0.36 (-0.15, 0.87)
3	0.39 (0.09, 0.70) ^*^	0.66 (0.15, 1.16) ^*^
4	0.57 (0.26, 0.88) ^*^	0.50 (0.03, 0.97) ^*^
5 +	0.95 (0.65, 1.26) ^*^	0.64 (0.16, 1.12) ^*^
***Outcome 2: Psychological burden***		
Count of chronic diseases (0–9)	0.46 (0.33, 0.58) ^*^	0.36 (0.17, 0.54) ^*^
Disease burden		
0–1	Ref	Ref
2	1.59 (0.83, 2.35) ^*^	1.39 (0.27, 2.50) ^*^
3	1.27 (0.56, 1.99) ^*^	0.86 (-0.20, 1.93)
4	1.52 (0.78, 2.25) ^*^	1.40 (0.38, 2.42) ^*^
5 +	2.68 (1.98, 3.39) ^*^	2.22 (1.19, 3.26) ^*^
***Outcome 3: Social burden***		
Count of chronic diseases (0–9)	0.13 (0.08, 0.18) ^*^	0.05 (-0.02, 0.11)
Disease burden		
0–1	Ref	Ref
2	0.11 (-0.17, 0.39)	0.21 (-0.19, 0.60)
3	0.28 (0.01, 0.55) ^*^	-0.14 (-0.53, 0.25)
4	0.47 (0.19, 0.74) ^*^	0.25 (-0.11, 0.62)
5 +	0.54 (0.27, 0.80) ^*^	0.15 (-0.22, 0.51)
***Outcome 4: Financial burden***		
Count of chronic diseases (0–9)	0.02 (0.01, 0.04) ^*^	0.02 (-0.01, 0.04)
Disease burden		
0–1	Ref	Ref
2	-0.01 (-0.12, 0.09)	-0.05 (-0.19, 0.09)
3	0.01 (-0.09, 0.10)	-0.08 (-0.22, 0.06)
4	0.05 (-0.05, 0.16)	-0.01 (-0.14, 0.12)
5 +	0.08 (-0.02, 0.18)	0.01 (-0.12, 0.14)
***Outcome 5: Caregiving gain***		
Count of chronic diseases (0–9)	0.01 (-0.02, 0.04)	-0.02 (-0.06, 0.02)
Disease burden		
0–1	Ref	Ref
2	0.09 (-0.07, 0.26)	-0.01 (-0.26, 0.24)
3	0.08 (-0.07, 0.24)	-0.03 (-0.27, 0.22)
4	0.04 (-0.12, 0.19)	-0.14 (-0.37, 0.09)
5 +	0.09 (-0.07, 0.25)	-0.06 (-0.29, 0.17)

*Note.* Abbreviations: *CI* confidence interval

^*^p < 0.05

^a^ Adjusted model was adjusted for care recipient’s gender, age, and race; caregiver’s gender, age, race, relationship to care recipient, education level, caregiving hours, and long-term caregiving, caregiving activities (help care recipient with ADL-IADL, health management, medical tasks), and formal and informal caregiving support

**Table 4:** Poisson regression result for physical burden score and comorbidities

	Beta coefficient (95% CI)	
	Unadjusted model	Adjusted model ^a^
Count of chronic diseases (0–9)	0.10 (0.07, 0.13) ^*^	0.06 (0.02, 0.11) ^*^
Disease burden		
0–1	Ref	Ref
2	0.09 (-0.10, 0.28)	0.19 (-0.09, 0.48)
3	0.19 (0.01, 0.36) ^*^	0.27 (0.002, 0.53) ^*^
4	0.31 (0.14, 0.49) ^*^	0.36 (0.09, 0.62) ^*^
5 +	0.45 (0.28, 0.62) ^*^	0.36 (0.10, 0.63) ^*^

*Note.* Abbreviations: *CI* confidence interval

^*^ *p* < 0.05

^a^ Adjusted model was adjusted for care recipient’s gender, age, and race; caregiver’s gender, age, race, relationship to care recipient, education level, caregiving hours, and long-term caregiving, caregiving activities (help care recipient with ADL-IADL, health management, medical tasks), and formal and informal caregiving support

### Association between latent class membership and caregiving burden and gain

In addition to the count of chronic conditions, we also measured comorbidity burden by dividing PWD into different comorbidity burden groups based on latent class analysis (LCA). To examine the patterns from the nine comorbid chronic conditions, we ran LCA from two- to five-classes. The two-class model yielded the optimal fit, as it has the lowest value on the Bayesian Information Criterion and the best interpretability (Supplementary Table S3). The two-class model divides care recipients into two classes—a low comorbidity burden class and a high comorbidity burden class. The largest portion of participants (69.0%) was assigned to the high comorbidity burden class. Caregivers in the high comorbidity class also has significantly higher average scores on their physical, psychological, and social burden (Supplementary Table S4).

After multivariable adjustment, caregivers of PWD in the high comorbidity burden class had a 0.46-point higher score of physical caregiving burden than those in the low comorbidity burden class (Table 5). There were no significant association between the latent class group of the care recipients and the psychological, social, financial burden, or gain that caregivers have (Table 5).

**Table 5:** Association between care recipient’s latent class group and caregiver’s burden

Outcomes: caregiving burden	Risk factor: high comorbidity burden class (ref. = low burden class)		
	Model 1^a^	Model 2^b^	Model 3^c^
	Beta coefficient (95% CI)		
Physical Burden	0.52 (0.30, 0.73) ^*^	0.46 (0.11, 0.80) ^*^	0.46 (0.13, 0.80) ^*^
Psychological Burden	0.60 (-0.10, 1.29)	0.40 (-0.30, 1.10)	0.31 (-0.43, 1.04)
Social Burden	0.37 (0.17, 0.57) ^*^	-0.02 (-0.29, 0.25)	-0.01 (-0.24, 0.22)
Financial Burden	0.03 (-0.04, 0.10)	0.01 (-0.08, 0.10)	0.01 (-0.09, 0.10)
Caregiving Gain	0.01 (-0.08, 0.11)	-0.01 (-0.13, 0.11)	-0.02 (-0.15, 0.13)

*Note.* Abbreviations: *CI* confidence interval

^*^ *p* < 0.05

^a^ Model 1 adjusted for care recipient’s gender, age, and race

^b^ Model 2 adjusted for care recipient’s gender, age, and race; and caregiver’s gender, age, race, relationship to care recipient, education level, caregiving hours, and long-term caregiving

^c^ Model 3 adjusted for care recipient’s gender, age, and race; caregiver’s gender, age, race, relationship to care recipient, education level, caregiving hours, and long-term caregiving, caregiving activities (help care recipient with ADL-IADL, health management, medical tasks), and formal and informal caregiving support

## Discussion

This study aimed to study the relationship between the comorbidity burden of older adults with dementia and their caregiver’s physical, psychological, social, and financial burden. We showed that a greater number of medical comorbidities is associated with a significant increase in the caregiver’s physical and psychological burden. Latent class analysis further revealed that caregivers of PWD in the high comorbidity burden group has significantly higher physical burden than caregivers of PWD in the low comorbidity burden group. However, despite experiencing higher physical and psychological burden, caregivers of PWD with more comorbidities did not experience significant changes in their social or financial burden or caregiving gain.

Our findings were in line with previous studies regarding the association between medical comorbidities and caregiving burden for PWD. Previous reports have shown that a higher number of medical comorbidities experienced by PWD is associated with a higher score on the Zarit Burden Interview for their caregivers, a composite score that measured the overall emotional and social burden of caregivers [15, 16]. Diabetes and osteoporosis were also shown to be the two conditions that are associated with a significantly higher caregiving emotional difficulty [12]. Our study broke down caregiving burden, a rather general concept, into four specific domains—physical, psychological, social, and financial, and measured each specific domain of caregiving burden based on multiple items from the NSOC survey to capture its complexity. We revealed that an increase in comorbidity burden is indeed associated with an increase in the physical and psychological burden of caregivers.

Such findings can be explained by both the increase in physical tasks and emotional stress for dementia caregivers brough by comorbidities, as well as the effectiveness of intervention programs that target the physical and emotional wellbeing of dementia caregivers. First, comorbidities among PWD, such as diabetes, osteoporosis, or cancer, occur with a high demand of everyday care [14], challenging symptom managements and treatments [26], more complications such as hypoglycemia and falls [27, 28], and limited support from healthcare providers [14]. These are all associated with more physical tasks to handle and more emotional stress for the caregiver to navigate through [12]. Second, caregiver support programs such as counseling and therapy sessions [29], self-care and healthy behavior education [30], yoga and meditation [31], leisure activities and exercises [32], and respite care services [33] were also shown to result in significantly lower cardiovascular disease risk [32], better sleep quality, improved physical health, better mood [30], less depression symptoms [29], and reduced stress [33]. The caregiving stress of dementia caregivers are thus indeed relieved by support programs targeting their physical and emotional well-being.

However, we did not find a significant association between comorbidities of the care recipients and the social or financial burden of their caregivers. One plausible explanation is that even though the social and financial costs of dementia caregiving are high, an increase in comorbidities may not be associated with additional social or financial strain. Given the high fixed cost of dementia caregiving that caregivers already paid by reduced social interactions, confined personal space [34], resignation from work, refusal of promotion opportunities [5, 35], etc., managing an additional comorbid chronic condition may no longer incur a substantial increase in additional burden. Furthermore, though there are various support programs that target the social and financial stress of dementia caregivers, previous literature showed limited efficacy of such program in improving caregivers’ wellbeing [36–39]. Therefore, future research is needed to investigate more into the mechanism behind whether and how comorbidity influences the social or financial burden of caregivers.

Caring for PWD with comorbidities can be challenging in many aspects; therefore, caregiver support programs are crucial in addressing what caregivers need the most. The result of this study shows that to best support dementia caregivers who care for PWD with comorbidities, support programs should specifically target their physical exhaustion and emotional stress. Such interventions could either address the care-recipients’ side by improving PWD’s healthcare experience when managing their multimorbidity or focus on the caregivers’ side by providing respite care services, psychotherapy, self-care education, skills training, etc., to relieve their physical and psychological burden.

Our study captured the complexity of PWD’s multimorbidity patterns and the multiple layers of caregiving burden. However, several limitations of this study should be noted. First, using a cross-sectional sample, we did not capture the impact of medical comorbidities on changes in caregiver burden over time. As past literature has made different proposals about how caregiving burden might decrease (as caregivers gradually adapt to the demand of their role) or increase (the longer they remains in the role, the greater caregivers are drained by the high demand) as time goes on [5], there might be a different pattern if this relationship were studied using longitudinal data. Second, for PWD with multiple caregivers, only their primary caregivers with the longest hours of caregiving are included in our sample, even though their secondary caregivers might also have considerable hours of caregiving. The existence of multiple caregivers might also affect the burden of each individual caregiver, yet this relationship was not fully investigated in our current study. Third, only nine major comorbid conditions measured in the NHATS interviews were included in our measure of PWD’s comorbidity burden. Other important comorbidities not included in this study, such as vision loss or hearing loss, might greatly affect the PWD’s communication capabilities and further complicate the level of caregiving burden. Fourth, for the nine comorbidities in this study, the specific symptoms or severity of each condition can greatly affect the comorbidity burden that a person with dementia bears, yet data about disease severity or symptoms were not recorded by NHATS and are thus not captured by our current investigation. Fifth, there might be other important aspects of caregiving burden that were not captured by the NSOC interviews and were thus not included in the current study. For example, financial burden of caregiving might also include the cost that a caregiver helps the care recipient pay for their medical care; however, since this specific aspect of financial burden was not individually surveyed in NSOC, it was not included in our current account of financial burden.

## Conclusions

Our study showed that caring for PWD with more multiple coexisting chronic conditions is indeed more stressful both physically and emotionally. However, no significant relationship is found between comorbidities of PWD and their caregiver’s social burden, financial burden, or gain. Therefore, to help relieve caregivers’ burden in the most effective way, community and healthcare facilities can develop programs for caregivers of PWD that target their physical and emotional wellbeing, such as healthy eating, yoga, meditation, regular breaks and exercises [40], counseling and therapy [41], or trainings to improve communication with PWD [26]. Future research could investigate more into the mechanisms that explain this relationship we found between dementia comorbidities and caregiving burden to develop guidance on the management of comorbid chronic conditions for PWD.

## Supplementary Information

**Additional file 1:** **Supplementary Table S1.** Weighted sample description by year interviewed (weighted *N* = 1,065). **Supplementary Table S2.** Fullregression results of associations between caregiving burden or gain and PWD’s comorbidities in the weighted sample. **Supplementary Table S3.** Latent class analysis model statistics. **Supplementary Table S4.** Caregiving burden scores by care recipient’s latent class groups.

## Data Availability

The datasets generated and analyzed during current study are publicly available at www.nhats.org/researcher/data-access [39–41].

## Abbreviations

aBIC: Adjusted Bayesian Information Criterion
ADL: Activities of daily living
AD8: The Eight-item Informant Interview to Differentiate Aging and Dementia
AIC: Akaike Information Criteria
BIC: Bayesian Information Criterion
CI: Confidence interval
GAD-2: Generalized Anxiety Disorder 2-item
IADL: Instrumental activities of daily living
LCA: Latent class analysis
LMR: Lo-Mendell-Rubin adjusted likelihood ratio test
NHATS: National Health and Aging Trends Study
NSOC: National Study of Caregiving
PHQ-2: Patient Health Questionnaire-2
PWD: Persons with dementia
SD: Standard Deviation
