# Supplementary material for: Association of dementia comorbidities with caregivers’ physical, psychological, social, and financial burden
Source: BMC Geriatr. 2023 Jan 31;23:60. doi: 10.1186/s12877-023-03774-9 (PMC9890694; doi:10.1186/s12877-023-03774-9)
Supplement: Supplementary file 1 — Additional file 1: Supplementary Table S1. Weighted sample description by year interviewed (weighted N = 1,065). Supplementary Table S2. Fullregression results of associations between caregiving burden or gain and PWD’s comorbidities in the weighted sample. Supplementary Table S3. Latent class analysis model statistics. Supplementary Table S4. Caregiving burden scores by care recipient’s latent class groups. [file 12877_2023_3774_MOESM1_ESM.docx]

**Additional File 1**

**Supplementary Table S1.** Weighted sample description by year interviewed (weighted *N* = 1,065)

| Year Interviewed | Total | 2011 | 2015 | 2017 | | Chi-square p-value |
| --- | --- | --- | --- | --- | --- | --- |
| *Care recipients* |  |  |  |  | |  |
| Sex, female (%) | 63.2 | 65.6 | 59.2 | 63.9 | | 0.156 |
| Race (%) |  |  |  |  | | 0.882 |
| White, non-Hispanic | 68.0 | 65.9 | 68.1 | 71.4 | |  |
| Black, non-Hispanic | 12.9 | 12.0 | 11.9 | 15.6 | |  |
| Other, non-Hispanic | 5.47 | 5.17 | 7.23 | 3.93 | |  |
| Hispanic | 13.6 | 16.9 | 12.8 | 9.15 | |  |
| Age (%) |  |  |  |  | | 0.175 |
| 65-74 years old | 14.3 | 13.5 | 18.8 | 10.6 | |  |
| 75-84 years old | 40.9 | 41.1 | 39.2 | 42.4 | |  |
| 85+ years old | 44.8 | 45.4 | 42.0 | 47.0 | |  |
| Number of chronic conditions (SD) | 3.33 (1.65) | 3.30 (1.65) | 3.40 (1.70) | 3.31 (1.59) | | 0.635 |
| *Caregivers* |  |  |  |  | |  |
| Sex, female (%) | 68.7 | 65.5 | 71.8 | 69.9 | | 0.188 |
| Age, over 65 years old (%) | 35.7 | 35.0 | 34.6 | 38.1 | | 0.951 |
| Race (%) |  |  |  |  | | 0.255 |
| White, non-Hispanic | 68.4 | 0 | 68.9 | 67.9 | |  |
| Black, non-Hispanic | 13.7 | 0 | 12.3 | 15.2 | |  |
| Other, non-Hispanic | 4.10 | 0 | 6.64 | 1.30 | |  |
| Hispanic | 13.8 | 0 | 12.3 | 15.5 | |  |
| Relationship to PLWD, spouse (%) | 19.1 | 20.9 | 20.3 | 14.8 | | 0.126 |
| Highest education, college or above (%) | 24.9 | 23.7 | 25.0 | 26.5 | | 0.254 |
| Self-rated health, poor or fair (%) | 20.5 | 24.2 | 17.0 | 18.7 | | 0.017 |
| Total (%) | 100 | 41.8 | 30.6 | 27.6 |  | |

*Note*. The first wave of NSOC in 2011 did not collect caregivers’ race data.

%: percentage coefficient

SD: mean coefficient, SD in parentheses

**Supplementary Table S2.** Full regression results of associations between caregiving burden or gain and PWD’s comorbidities in the weighted sample.

|  | Beta coefficient (95% CI) | | | |
| --- | --- | --- | --- | --- |
|  | Unadjusted, Count of chronic diseases | Adjusted, Count of chronic diseases ^a^ | Unadjusted, Disease burden groups | Adjusted, Disease burden groups ^a^ |
| ***Outcome 1: Physical Burden*** | | | | |
| Count of chronic diseases | 0.18 (0.13, 0.23) * | 0.11 (0.03, 0.20) * |  |  |
| Disease Burden |  |  |  |  |
| 0-1 |  |  | Ref. | Ref. |
| 2 |  |  | 0.36 (0.04, 0.69) | 0.36 (-0.15, 0.87) |
| 3 |  |  | 0.40 (0.08. 0.70) | 0.66 (0.15, 1.16) * |
| 4 |  |  | 0.57 (0.26, 0.88) | 0.50 (0.03, 0.97) * |
| 5+ |  |  | 0.95 (0.65, 1.26) | 0.64 (0.16, 1.12) * |
| PWD gender, female |  | 0.38 (0.05, 0.70) * |  | 0.40 (0.07, 0.72) * |
| PWD age |  |  |  |  |
| 65-74 years old |  | Ref. |  | Ref. |
| 75-84 years old |  | -0.36 (-0.46, 0.38) |  | -0.00 (-0.43, 0.43) |
| 85+ years old |  | 0.12 (-0.32, 0.56) |  | 0.16 (-0.29, 0.60) |
| PWD race |  |  |  |  |
| White, non-Hispanic |  | Ref. |  | Ref. |
| Black, non-Hispanic |  | 0.21 (-1.31, 1.73) |  | 0.09 (-1.43, 1.61) |
| Other, non-Hispanic |  | -0.05 (-1.02, 0.92) |  | -0.10 (-1.08, 0.88) |
| Hispanic |  | 0.16 (-0.66, 0.98) |  | 0.26 (-0.57, 1.09) |
| Caregiver gender, female |  | 0.35 (0.03, 0.66) * |  | 0.35 (0.03, 0.68) * |
| Caregiver age, over 65 |  | 0.23 (-0.15, 0.61) |  | 0.18 (-0.20, 0.56) |
| Caregiver race |  |  |  |  |
| White, non-Hispanic |  | Ref. |  | Ref. |
| Black, non-Hispanic |  | -0.34 (-1.85, 1.16) |  | -0.23 (-1.73, 1.28) |
| Other, non-Hispanic |  | 0.17 (-0.87, 1.21) * |  | 0.22 (-0.84, 1.27) |
| Hispanic |  | -0.69 (-1.49. 0.11) |  | -0.79 (-1.60, 0.02) |
| Relationship to PLWD, spouse |  | 0.48 (-0.04, 1.01) |  | 0.54 (0.01, 1.07) * |
| Highest education, college or above |  | -0.35 (-0.70, -0.01) * |  | -0.39 (-0.74, -0.05) * |
| Caregiving hours |  | 0.00 (-0.00, 0.00) |  | 0.00 (-0.00, 0.00) |
| Long term caregiving, over 5 years |  | 0.26 (-0.03, 0.54) |  | 0.25 (-0.04, 0.54) |
| Caregiving tasks: ADL/IADL |  | 0.03 (-0.09, 0.16) |  | 0.03 (-0.10, 0.16) |
| Caregiving tasks: medical |  | -0.00 (-0.07, 0.07) |  | 0.00 (-0.06, 0.07) |
| Caregiving support |  | 0.00 (-0.12. 0.13) |  | -0.01 (-0.13, 0.12) |
| Constant | 0.99 (0.79, 1.19) * | 0.18 (-0.63, 0.99) | 1.08 (0.83, 1.32) * | 0.09 (-0.76, 0.93) |
| ***Outcome 2: Psychological Burden*** | | | | |
| Count of chronic diseases | 0.46 (0.33, 0.58) * | 0.36 (0.17, 0.54) * |  |  |
| Disease Burden |  |  |  |  |
| 0-1 |  |  | Ref. | Ref. |
| 2 |  |  | 1.59 (0.83, 2.34) * | 1.39 (0.27, 2.50) * |
| 3 |  |  | 1.27 (0.56, 1.99) * | 0.86 (-0.20, 1.93) |
| 4 |  |  | 1.52 (0.78, 2.25) * | 1.40 (0.38. 2.42) * |
| 5+ |  |  | 2.68 (1.98, 3.39) * | 2.22 (1.19, 3.26) * |
| PWD gender, female |  | 0.05 (-0.64, 0.74) |  | 0.05 (-0.65, 0.74) |
| PWD age |  |  |  |  |
| 65-74 years old |  | Ref. |  | Ref. |
| 75-84 years old |  | 0.23 (-0.68, 1.13) |  | 0.36 (-0.55, 1.27) |
| 85+ years old |  | -0.53 (-1.48, 0.43) |  | -0.38 (-1.33, 0.58) |
| PWD race |  |  |  |  |
| White, non-Hispanic |  | Ref. |  | Ref. |
| Black, non-Hispanic |  | 0.92 (-2.42, 4.25) |  | 0.81 (-2.51, 4,14) |
| Other, non-Hispanic |  | -0.63 (2.67, 1.42) |  | -0.39 (-2.44, 1.67) |
| Hispanic |  | -0.02 (-1.72, 1.68) |  | -0.12 (-1.84, 1.60) |
| Caregiver gender, female |  | 0.57 (-0.11. 1.24) |  | 0.51 (-0.17, 1.19) |
| Caregiver age, over 65 |  | -0.65 (-1.48, 0.19) |  | -0.68 (-1.52, 0.17) |
| Caregiver race |  |  |  |  |
| White, non-Hispanic |  | Ref. |  | Ref. |
| Black, non-Hispanic |  | -1.36 (-4.64, 1.91) |  | -1.26 (-4.52, 2.01) |
| Other, non-Hispanic |  | 2.49 (0.25, 4.73) * |  | 2.21 (-0.05, 2.37) |
| Hispanic |  | -0.41 (-2.07, 1.25) |  | -0.31 (-1.99, 1.37) |
| Relationship to PLWD, spouse |  | 1.22 (0.07, 2.37) * |  | 1.21 (0.05, 2.37) * |
| Highest education, college or above |  | -0.08 (-0.80, 0.65) |  | -0.09 (-0.82, 0.64) |
| Caregiving hours |  | 0.00 (-0.00, 0.00) |  | 0.00 (-0.00, 0.00) |
| Long term caregiving, over 5 years |  | 0.37 (-0.25, 0.98) |  | 0.31 (-0.30, 0.93) |
| Caregiving tasks: ADL/IADL |  | 0.25 (-0.03, 0.53) |  | 0.29 (0.01, 0.57) * |
| Caregiving tasks: medical |  | 0.14 (0.002, 0.28) * |  | 0.11 (-0.03, 0.25) |
| Caregiving support |  | -0.39 (-0.66, -0.12) * |  | -0.41 (-0.68, -0.15) * |
| Constant | 3.17 (2.70, 3.65) * | 1.68 (-0.11. 3.47) | 3.14 (2.57, 3.71) * | 1.51 (-0.33, 3.36) |
| ***Outcome 3: Social Burden*** | | | | |
| Count of chronic diseases | 0.13 (0.08, 0.18) * | 0.05 (-0.02, 0.11) |  |  |
| Disease Burden |  |  |  |  |
| 0-1 |  |  | Ref. | Ref. |
| 2 |  |  | 0.11 (-0.18, 0.39) | 0.21 (-0.19, 0.60) |
| 3 |  |  | 0.28 (0.01, 0.55) * | -0.14 (-0.53, 0.25) |
| 4 |  |  | 0.47 (0.19, 0.74) * | 0.25 (-0.11, 0.62) |
| 5+ |  |  | 0.54 (0.27, 0.80) * | 0.15 (-0.22, 0.51) |
| PWD gender, female |  | 0.03 (-0.22, 0.28) |  | 0.02 (-0.23, 0.27) |
| PWD age |  |  |  |  |
| 65-74 years old |  | Ref. |  | Ref. |
| 75-84 years old |  | 0.67 (0.35, 1.00) * |  | 0.69 (0.36, 1.02) * |
| 85+ years old |  | 0.50 (0.17, 0.85) * |  | 0.54 (0.20, 0.88) * |
| PWD race |  |  |  |  |
| White, non-Hispanic |  | Ref. |  | Ref. |
| Black, non-Hispanic |  | -0.60 (-1.78, 0.58) |  | -0.61 (-1.78, 0.57) * |
| Other, non-Hispanic |  | 1.07 (0.31, 1.82) * |  | 1.17 (0.42, 1.93) * |
| Hispanic |  | 0.01 (-0.31, 1.82) |  | -0.06 (-0.70, 0.58) |
| Caregiver gender, female |  | 0.12 (-0.12, 0.37) |  | 0.12 (-0.12, 0.37) |
| Caregiver age, over 65 |  | 0.09 (-0.20, 0.38) |  | 0.11 (-0.19, 0.40) |
| Caregiver race |  |  |  |  |
| White, non-Hispanic |  | Ref. |  | Ref. |
| Black, non-Hispanic |  | 0.56 (-0.60, 1.73) |  | 0.58 (-0.58, 1.74) |
| Other, non-Hispanic |  | -0.69 (-1.49, 0.11) |  | -0.87 (-1.68, -0.05) |
| Hispanic |  | 0.17 (-0.45, 0.78) |  | 0.26 (-0.36, 0.89) |
| Relationship to PLWD, spouse |  | -0.04 (-0.45, 0.36) |  | -0.04 (-0.44, 0.37) |
| Highest education, college or above |  | 0.06 (-0.20, 0.33) |  | 0.06 (-0.21, 0.33) |
| Caregiving hours |  | 0.00 (0.00, 0.00) |  | 0.00 (0.00, 0.00) |
| Long term caregiving, over 5 years |  | -0.23 (-0.45, -0.01) * |  | -0.24 (-0.46, -0.01) |
| Caregiving tasks: ADL/IADL |  | 0.09 (-0.02, 0.19) |  | 0.10 (0.002, 0.20) * |
| Caregiving tasks: medical |  | 0.13 (0.08, 0.18) * |  | 0.13 (0.08, 0.18) * |
| Caregiving support |  | 0.02 (-0.07, 0.12) |  | 0.02 (-0.08, 0.12) |
| Constant | 0.35 (0.17, 0.53) * | -1.32 (-1.95, -0.69) * | 0.47 (0.25, 0.69) * | -1.35 (-2.00, -0.70) * |
| ***Outcome 4: Financial Burden*** | | | | |
| Count of chronic diseases | 0.02 (0.01, 0.04) * | 0.02 (-0.01, 0.04) |  |  |
| Disease Burden |  |  |  |  |
| 0-1 |  |  | Ref. | Ref. |
| 2 |  |  | -0.01 (-0.12. 0.09) | -0.05 (-0.19, 0.09) |
| 3 |  |  | 0.01 (-0.09, 0.11) | -0.08 (-0.22, 0.06) |
| 4 |  |  | 0.05 (-0.05, 0.16) | -0.01 (-0.14, 0.12) |
| 5+ |  |  | 0.08 (-0.02, 0.18) | 0.01 (-0.12, 0.14) |
| PWD gender, female |  | 0.03 (-0.06, 0.12) |  | 0.02 (-0.07, 0.11) |
| PWD age |  |  |  |  |
| 65-74 years old |  | Ref. |  | Ref. |
| 75-84 years old |  | 0.18 (0.06, 0.29) |  | 0.17 (0.06, 0.29) * |
| 85+ years old |  | 0.15 (0.03, 0.27) |  | 0.15 (0.02, 0.27) * |
| PWD race |  |  |  |  |
| White, non-Hispanic |  | Ref. |  | Ref. |
| Black, non-Hispanic |  | 0.13 (-0.30, 0.55) |  | 0.12 (-0.30, 0.54) |
| Other, non-Hispanic |  | 0.51 (0.24, 0.78) * |  | 0.52 (0.25, 0.79) * |
| Hispanic |  | -0.03 (-0.25, 0.20) |  | -0.05 (-0.28, 0.18) |
| Caregiver gender, female |  | 0.08 (-0.01, 0.17) |  | 0.08 (-0.01, 0.17) |
| Caregiver age, over 65 |  | -0.19 (-0.29, -0.08) |  | -0.18 (-0.28, -0.07) * |
| Caregiver race |  |  |  |  |
| White, non-Hispanic |  | Ref. |  | Ref. |
| Black, non-Hispanic |  | -0.15 (-0.56, 0.27) |  | -0.14 (-0.09, 0.36) |
| Other, non-Hispanic |  | -0.08 (-0.37, 0.21) |  | -0.09 (-0.39, 0.20) |
| Hispanic |  | 0.11 (-0.11, 0.33) |  | 0.13 (-0.09, 0.36) |
| Relationship to PLWD, spouse |  | -0.02 (-0.16, 0.13) |  | -0.03 (-0.17, 0.12) |
| Highest education, college or above |  | 0.12 (0.02, 0.21) * |  | 0.12 (0.02, 0.22) * |
| Caregiving hours |  | 0.00 (-0.00, 0.00) |  | 0.00 (-0.00, 0.00) |
| Long term caregiving, over 5 years |  | -0.00 (-0.08, 0.08) |  | -0.00 (-0.08, 0.08) |
| Caregiving tasks: ADL/IADL |  | 0.05 (0.02, 0.09) * |  | 0.06 (0.02, 0.09) * |
| Caregiving tasks: medical |  | 0.01 (-0.01, 0.03) |  | 0.01 (-0.01, 0.03) |
| Caregiving support |  | -0.02 (-0.05, 0.02) |  | -0.02 (-0.05, 0.02) |
| Constant | 0.15 (0.08, 0.22) * | -0.28 (-0.51, -0.06) * | 0.20 (0.12, 0.28) * | -0.22 (-0.46, 0.01) |
| ***Outcome 5: Caregiving Gain*** | | | | |
| Count of chronic diseases | 0.01 (-0.02, 0.04) * | -0.02 (-0.06, 0.02) |  |  |
| Disease Burden |  |  |  |  |
| 0-1 |  |  | Ref. | Ref. |
| 2 |  |  | 0.09 (-0.07, 0.26) | -0.01 (-0.26, 0.24) |
| 3 |  |  | 0.08 (-0.08, 0.24) | -0.03 (-0.27, 0.22) |
| 4 |  |  | 0.04 (-0.13, 0.20) | -0.14 (-0.37, 0.09) |
| 5+ |  |  | 0.09 (-0.07, 0.25) | -0.06 (-0.29, 0.13) |
| PWD gender, female |  | 0.17 (0.01, 0.32) * |  | 0.16 (0.01, 0.32) * |
| PWD age |  |  |  |  |
| 65-74 years old |  | Ref. |  | Ref. |
| 75-84 years old |  | -0.18 (-0.38, 0.02) |  | -0.16 (-0.37, 0.04) |
| 85+ years old |  | -0.08 (-0.29, 0.13) |  | -0.08 (-0.29, 0.13) |
| PWD race |  |  |  |  |
| White, non-Hispanic |  | Ref. |  | Ref. |
| Black, non-Hispanic |  | 0.22 (-0.51, 0.96) |  | 0.23 (-0.49, 0.97) |
| Other, non-Hispanic |  | 0.06 (-0.41, 0.53) |  | 0.05 (-0.43, 0.97) |
| Hispanic |  | 0.11 (-0.29, 0.50) |  | 0.10 (-0.30, 0.50) |
| Caregiver gender, female |  | 0.09 (-0.06, 0.24) |  | 0.08 (-0.08, 0.23) |
| Caregiver age, over 65 |  | -0.05 (-0.23, 0.13) |  | -0.05 (-0.24, 0.13) |
| Caregiver race |  |  |  |  |
| White, non-Hispanic |  | Ref. |  | Ref. |
| Black, non-Hispanic |  | 0.05 (-0.68, 0.77) |  | 0.03 (-0.69, 0.76) |
| Other, non-Hispanic |  | -0.16 (-0.66, 0.34) |  | -0.13 (-0.64, 0.38) |
| Hispanic |  | -0.21 (-0.59, 0.18) |  | -0.20 (-0.59, 0.19) |
| Relationship to PLWD, spouse |  | 0.07 (-0.18, 0.32) |  | 0.07 (-0.19, 0.32) |
| Highest education, college or above |  | -0.32 (-0.49, -0.16) |  | -0.32 (-0.49, -0.15) * |
| Caregiving hours |  | -0.00 (-0.00, 0.00) |  | -0.00 (-0.00, 0.00) |
| Long term caregiving, over 5 years |  | -0.01 (-0.15, 0.13) |  | -0.01 (-0.15, 0.13) |
| Caregiving tasks: ADL/IADL |  | 0.03 (-0.04, 0.09) |  | 0.03 (-0.04, 0.09) |
| Caregiving tasks: medical |  | 0.03 (0.00, 0.06) |  | 0.03 (-0.00, 0.06) |
| Caregiving support |  | -0.04 (-0.10, 0.03) |  | -0.03 (-0.09, 0.03) |
| Constant | 3.55 (3.45, 3.69) * | 3.55 (3.16, 3.94) * | 3.52 (3.40, 3,65) * | 3.56 (3.15, 3.96) |

*Note.* Abbreviations: CI, confidence interval.

^*^ *p* < 0.05

^a^ Adjusted Model was adjusted for care recipient’s gender, age, and race; caregiver’s gender, age, race, relationship to care recipient, education level, caregiving hours, and long-term caregiving, caregiving activities (help care recipient with ADL-IADL, health management, medical tasks), and formal and informal caregiving support.

**Supplementary Table S3.** Latent class analysis model statistics.

|  | 2-Class | 3-Class | 4-Class | 5-Class |
| --- | --- | --- | --- | --- |
| Bayesian Information Criterion | 11124.1 | 11141.5 | 11172.9 | 11211.1 |

*Note.* The two-class model has the lowest Bayesian Information Criterion value.

**Supplementary Table S4.** Caregiving burden scores by care recipient’s latent class groups.

| Latent class membership | High comorbidity burden class | Low comorbidity burden class | P-value |
| --- | --- | --- | --- |
| Physical burden (0-5) | 1.86 | 1.12 | 0.001 |
| Psychological burden (0-18) | 5.15 | 3.87 | 0.015 |
| Social burden (0-5) | 0.96 | 0.49 | 0.007 |
| Financial burden (0-2) | 0.24 | 0.21 | 0.609 |
| Gain (0-4) | 3.59 | 3.59 | 0.967 |

*Note.* Coefficients show the mean outcome scores. P-value comes from comparison of means between the two groups.
